# Creating National Air Pollution Models for Population Exposure Assessment in Canada

**DOI:** 10.1289/ehp.1002976

**Published:** 2011-03-31

**Authors:** Perry Hystad, Eleanor Setton, Alejandro Cervantes, Karla Poplawski, Steeve Deschenes, Michael Brauer, Aaron van Donkelaar, Lok Lamsal, Randall Martin, Michael Jerrett, Paul Demers

**Affiliations:** 1 School of Population and Public Health, University of British Columbia, Vancouver, British Columbia, Canada; 2 Department of Geography, University of Victoria, Victoria, British Columbia, Canada; 3 Department of Geography, and; 4 School of Environmental Health, University of British Columbia, Vancouver, British Columbia, Canada; 5 Department of Physics and Atmospheric Science, Dalhousie University, Halifax, Nova Scotia, Canada; 6 Harvard-Smithsonian Center for Astrophysics, Cambridge, Massachusetts, USA; 7 School of Public Health, Division of Environmental Health Science, University of California–Berkeley, Berkeley, California, USA; 8 Occupational Cancer Research Centre, Cancer Care Ontario, Toronto, Ontario, Canada

**Keywords:** air pollution, Canada, fixed-site monitors, gradients, land use regression, population exposure assessment, satellite data

## Abstract

Background: Population exposure assessment methods that capture local-scale pollutant variability are needed for large-scale epidemiological studies and surveillance, policy, and regulatory purposes. Currently, such exposure methods are limited.

Methods: We created 2006 national pollutant models for fine particulate matter [PM with aerodynamic diameter ≤ 2.5 μm (PM_2.5_)], nitrogen dioxide (NO_2_), benzene, ethylbenzene, and 1,3-butadiene from routinely collected fixed-site monitoring data in Canada. In multiple regression models, we incorporated satellite estimates and geographic predictor variables to capture background and regional pollutant variation and used deterministic gradients to capture local-scale variation. The national NO_2_ and benzene models are evaluated with independent measurements from previous land use regression models that were conducted in seven Canadian cities. National models are applied to census block-face points, each of which represents the location of approximately 89 individuals, to produce estimates of population exposure.

Results: The national NO_2_ model explained 73% of the variability in fixed-site monitor concentrations, PM_2.5_ 46%, benzene 62%, ethylbenzene 67%, and 1,3-butadiene 68%. The NO_2_ model predicted, on average, 43% of the within-city variability in the independent NO_2_ data compared with 18% when using inverse distance weighting of fixed-site monitoring data. Benzene models performed poorly in predicting within-city benzene variability. Based on our national models, we estimated Canadian ambient annual average population-weighted exposures (in micrograms per cubic meter) of 8.39 for PM_2.5_, 23.37 for NO_2_, 1.04 for benzene, 0.63 for ethylbenzene, and 0.09 for 1,3-butadiene.

Conclusions: The national pollutant models created here improve exposure assessment compared with traditional monitor-based approaches by capturing both regional and local-scale pollution variation. Applying national models to routinely collected population location data can extend land use modeling techniques to population exposure assessment and to informing surveillance, policy, and regulation.

Predicting air pollution concentrations at resolutions capable of capturing local-scale pollutant gradients over large geographical areas is becoming increasingly important in multicity and national health studies; in population exposure assessment; and in support of policy, surveillance, and regulatory initiatives. Currently, fixed-site government monitors are the foundation of these activities; however, because of siting criteria, such monitors may fail to fully capture local-scale pollutant variability. In addition, the number of monitors and their spatial distribution may be limited, as is the case in Canada. At present, few methodologies are available that adequately capture local-scale pollutant variability at a national scale when monitor density, distribution, or siting is suboptimal.

A number of approaches may be used to model air pollution over large areas, including interpolation of fixed-site government monitoring data, dispersion modeling, satellite remote sensing, land use regression (LUR), and proximity and deterministic methods. Each approach, however, has inherent limitations that restrict its use for producing local-scale pollution estimates. Interpolation of fixed-site air pollution monitoring data has typically been used to predict pollution concentrations across large areas (Beelen et al. 2009), with recent interest directed towards kriging methods and spatial smoothing with geographic covariates (Beelen et al. 2009; Hart et al. 2009; Yanosky et al. 2008). Fixed-site monitors may not capture entire populations, and measurements typically represent regional and between-city pollution differences due to monitor siting criteria, which prevent monitors from being placed in proximity to major roads and other pollution sources. Dispersion models also exist for large geographical areas and have been incorporated into regulatory and epidemiological studies of air pollution (Cyrys et al. 2005; Nafstad et al. 2003). Importantly, the resolutions of pollutant estimates from dispersion models over large geographical areas are typically restricted, for example, to 1 or 3 km^2^ (Jerrett et al. 2005). Satellite remote sensing is a new methodology available to predict air pollution concentrations over large geographic areas, and a number of studies have evaluated different remotely sensed concentrations of fine particulate matter [PM with aerodynamic diameter ≤ 2.5 μm (PM_2.5_)] (e.g., van Donkelaar et al. 2010) and gaseous pollutants (Martin 2008) and found moderate to good associations with ground-level monitoring data. Currently, the resolution of satellite data limits their use to representing regional pollution concentrations, but indicators of local air pollution may be used in concert to improve the spatial resolution of predictions (Liu et al. 2009). LUR approaches have been used extensively to predict within-city pollutant concentrations of nitrogen dioxide (NO_2_) and PM_2.5_ (for review, see Hoek et al. 2008), but to a lesser extent for volatile organic compounds (VOCs). However, the approach is well suited to modeling pollutants that exhibit significant spatial variation, especially traffic-related VOCs (Atari and Luginaah 2009; Mukerjee et al. 2009; Smith et al. 2006; Su et al. 2010; Wheeler et al. 2008). The city-by-city approach in which LUR models are created is costly, and integration and interpretation across multiple city models is difficult. Simple proximity and deterministic approaches have also been widely used as surrogates for exposure to vehicle and industrial sources, specifically in epidemiological studies; yet, such measures in isolation are often poor surrogates for exposure. To date, few population exposure assessments have incorporated multiple sources of data, specifically satellite pollutant estimates, LUR modeling of geographic characteristics, and information on proximity and pollution gradients, to estimate local-scale air pollution concentrations at a national scale.

Here we report a modeling initiative to produce 2006 national PM_2.5_, NO_2_, benzene, ethylbenzene, and 1,3-butadiene models for Canada that capture local-scale pollutant variability and apply these models to routinely collected population location data to calculate population exposures. This research is part of Carex Canada, a national surveillance initiative designed to estimate the number of Canadians potentially exposed to known or suspected environmental and occupational carcinogens (Carex Canada 2011). This research adds to the literature on air pollution modeling and exposure assessment by creating national LUR models from fixed-site monitoring data; incorporating various predictor data sets and methods to capture the different scales of pollution sources; and extending LUR modeling techniques to population exposure assessment and to informing surveillance, policy, and regulation.

## Materials and Methods

*Pollutant modeling approach.* Models were developed in two stages using different predictor variables and methodology to capture background, regional, and local-scale pollution variation. First, for each National Air Pollution Surveillance (NAPS) fixed-site monitoring station, we derived satellite-based estimates (PM_2.5_ and NO_2_ only) and geographic variables (e.g., road length, population density, proximity to large emitters) using ArcGIS (version 9.3; ESRI, Redland, CA, USA). We used forward stepwise regression to develop LUR models and retained variables that corresponded to hypothesized effect directions; we maximized the sums of squares explained by Akaike’s information criterion. Spatial autocorrelation was also evaluated using the Moran’s *I* statistic in ArcGIS. We sought to develop parsimonious models rather than traditional predictive models that maximize prediction but make interpretation of individual variable contributions difficult. Only variables significant at the *p* < 0.05 level were included in the final models. As expected, NAPS monitoring locations in Canada did not display sufficient variability to estimate model coefficients for important local-scale parameters, such as proximity to major roadways, because of monitor siting. Thus, local-scale predictors were underpowered in the LUR modeling approach.

In the second stage, we conducted comprehensive literature reviews to identify deterministic factors to represent local-scale gradients in pollutant concentrations associated with specific sources (i.e., highways, major roads, gas stations). For each pollutant, we identified concentrations near these selected sources in relation to local background levels and developed deterministic multipliers with distance decay rates (together referred to as gradients in this paper) to apply to the background and regional concentrations predicted by our LUR models. All statistical analyses were conducted using SAS (version 9.1; SAS Institute Inc., Cary, NC, USA).

**Figure 1:**
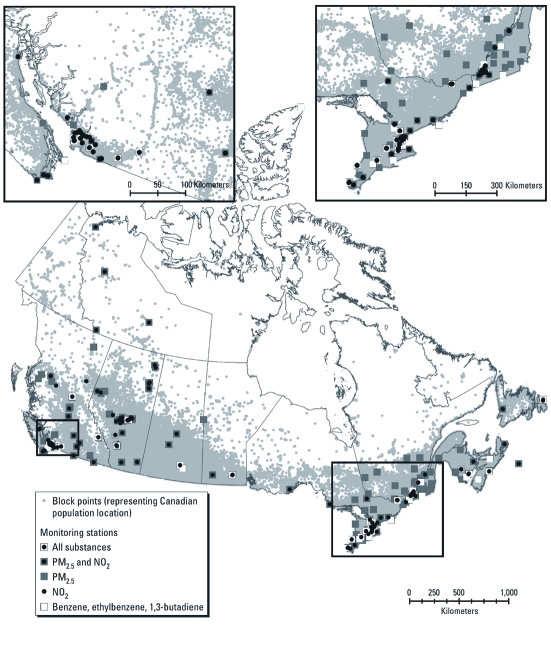
Location of NAPS monitors that were used to create national PM_2.5_, NO_2_, benzene, ethylbenzene, and 1,3-butadiene models.

*Air quality data.* Annual average concentrations of PM_2.5_ (177 monitoring stations), NO_2_ (134 monitors), and benzene, ethylbenzene, and 1,3-butadiene (53 monitors) were calculated using data from unique NAPS monitoring sites that were operating during 2006 (see Figure 1). Continuous monitoring data from a given monitor were included if at least 50% of hourly observations were available for a 24-hr period and at least 50% of days were available in a month. Monthly averages from filter-based PM_2.5_ measurements required a minimum of three of five valid measurements per month. Annual averages for 2006 were not calculated for individual monitors unless there were at least 6 months of complete data with one valid month per quarter.

NAPS includes different monitor types for PM_2.5_, including tapered element oscillating microbalances (TEOMs), dichotomous partisol samplers (Thermo Fisher Scientific Inc., Waltham, MA, USA), and beta-attenuation mass monitors (Met One Instruments Inc., Grants Pass, OR, USA). Multiple monitors are often present at one location, and our comparative analysis found differences in levels measured by TEOMs, which are known to underpredict PM_2.5_ because of nitrate evaporation (Dann T, personal communication). We therefore selected other monitor types when they were available at the same location. Those stations with only TEOMs available were adjusted based on yearly calibration between collocated dichotomous and TEOM monitors during 2006 [*n =* 14, dichotomous = 1.640 + 1.089 × (TEOM), *R*^2^ = 0.89, *p* < 0.001]. NO_2_, benzene, ethylbenzene, and 1,3-butadiene were measured using standard methods (NAPS 2004).

*Predictor variables.* PM_2.5_ and NO_2_ satellite data. Canada-wide concentrations of PM_2.5_ and NO_2_ were estimated using satellite atmospheric composition data combined with local, coincident scaling factors from a chemical transport model [Goddard Earth Observing System (GEOS)-Chem 2011]. Ground-level PM_2.5_ estimates were derived from aerosol optical depth data from the Terra satellite [National Aeronautics and Space Administration (NASA) 2011b], in combination with output from GEOS-Chem simulations to estimate the relationship between aerosol optical depth over the atmospheric column and ground-level PM_2.5_ (van Donkelaar et al. 2010). Ground-level NO_2_ concentrations were estimated from tropospheric NO_2_ columns retrieved from the ozone monitoring instrument on the Aura satellite (NASA 2011a); GEOS-Chem was also used to calculate the relationship between the NO_2_ column and ground-level concentration (Lamsal et al. 2008). Both PM_2.5_ and NO_2_ were estimated at a 0.1 × 0.1° resolution (~ 10 × 10 km). Estimates for PM_2.5_ were calculated from 2001–2006 data to ensure sufficient observations. For NO_2_ estimates, we used data from 2005 and 2006, because ozone monitoring instrument measurements began in late 2004.

Geographic data. We modeled regional pollutant variation using geographic predictor variables potentially relevant to pollutant sources, emissions, and dispersion. To capture varying spatial influences of predictors, all variables were calculated for circular buffer distances ranging from 50 m to 50 km. Classes of variables included population density derived from census block-face points (Statistics Canada 2006); 1-km land use classifications (Global Land Cover Characterization 2008); high-resolution (30 m) land-use classifications (DMTI Spatial Inc., Markham, Ontario, Canada); sources of large industrial emissions from the Canadian National Pollutant Release Inventory (NPRI; Environment Canada 2010); small point source locations extracted from the Dun and Bradstreet (D&B) Selectory database of businesses (Hoovers, Austin, TX, USA) in Canada; length of and distance to specific road classifications using the DMTI Spatial road network, such as freeway, highway, major road, and minor road (DMTI Spatial Inc.); length and density of railroads; elevation; and meteorological variables (precipitation and temperature). Any geographic variables with > 30% zero values—those with no predictive features in proximity to a monitor—were recoded as binary (i.e., present/absent). In total, 10 variable classes and 270 buffer-specific variables were explored in the LUR models.

Deterministic gradients. Gradients were developed with a focus on mobile sources and gas stations. We conducted a comprehensive literature review of published studies to identify the distance from sources at which pollutant concentrations typically return to background levels, and an expected ratio of near-source pollutant levels compared with background pollutant levels for each source and pollutant. We searched PubMed (2010), Web of Science (Thomson Reuters 2010), and Google Scholar (2010) using a range of keywords to identify studies with measurements of pollutant gradients. Studies varied widely in terms of location, date, methods, duration of measures, number of samples, and definition of near source and background. We developed linear gradients using the steepest portion of the exponential decay curves typically found in the literature, as the tails of the decay functions were very sensitive to local parameters. Gradients were also selected to represent Canadian conditions. Table 1 summarizes the gradients developed for Canada and applied to the LUR models.

**Table 1:** PM_2.5_, NO_2_, benzene, ethylbenzene, and 1,3-butadiene gradients determined from the literature and incorporated with national LUR model predictions.

Substance	Source	Increase at source	Gradient distance (m)
PM_2.5_		Highway		1.25*a*		75*b*
		Major road		1.1*a*		75*b*
NO_2_		Highway		1.65*a*		300*c*
		Major road		1.2*a*		100*c*
Benzene		Gas station		6.5*d*		100*d*
		Highway/major road		3.25*e*		50*f*
		Local road		1.5*e*		50*f*
Ethylbenzene		Highway		3.7*g*		300*h*
		Major road		2.2*g*		300*h*
		Local road		1.4*g*		300*h*
1,3-Butadiene		Highway		4*i*		75*i*
**a**Smargiassi et al. (2005). **b**Beckerman et al. (2008), Hitchins et al. (2000), Roorda-Knape et al. (1998), Tiitta et al. (2002). **c**Beckerman et al. (2008), Gilbert et al. (2003, 2007), Roorda-Knape et al. (1998), Su et al. (2009). **d**Karakitsios et al. (2007). **e**Hellén et al. (2006), Parra et al. (2009), Thorsson and Eliasson (2006), Vardoulakis et al. (2002). **f**Beckerman et al. (2008), Thorsson and Eliasson (2006), Venkatram et al. (2009). **g**Parra et al. (2009), Roukos et al. (2009), Wang and Zhao (2008). **h**Wang and Zhao (2008). **i**Venkatram et al. (2009).						

To identify the distance of each NAPS monitor from the nearest highway, major road, local urban road, and gas station, we used DMTI road network data and D&B commercial data for point sources. If a monitor was close enough to one of these features for the source to influence pollutant levels, we modified the corresponding LUR model results (not including point source industrial variables) to account for the deterministic gradients. For example, based on our review of the literature, we assumed that NO_2_ concentrations at the side of a highway would be 1.65 times higher than LUR-based background concentrations but consistent with background levels 300 m from the highway; this assumption resulted in a distance decay rate of 0.33% per meter that was applied to the model to estimate NO_2_ levels within the 300-m gradient buffer.

*Model evaluation.* We used three approaches for model evaluation. Due to the small number of NAPS monitoring stations for PM_2.5_, NO_2_, benzene, ethylbenzene, and 1,3-butadience, we did not leave out a percentage for independent postmodel evaluation, because we wanted to capture the greatest range of model predictors possible. Therefore, we first evaluated all LUR models using a bootstrap approach to determine the sensitivity of model prediction and parameter estimates to monitor sampling. Random selection of monitors was conducted, with replacement, and variable coefficients and model *R*^2^ values were recorded from the new full sample. This was repeated for 10,000 iterations to estimate the 95% confidence interval (CI) for overall model prediction and individual variable coefficients. Next, we conducted a leave-one-out analysis where each LUR model was repeatedly parameterized on *n* – 1 data points and then used to predict the excluded monitor measurement. The mean differences between the predicted and measured values were used to estimate model error.

Finally, we evaluated the NO_2_ and benzene LUR models, with and without gradients, against independent data (35–196 monitoring sites per city) previously collected for LUR models in seven Canadian cities (for a full description of data collection and modeling see Allen et al. 2010; Atari and Luginaah 2009; Crouse et al. 2009; Henderson et al. 2007; Jerrett et al. 2007; Su et al. 2010). Briefly, in each city, monitoring took place over a 2-week period; data from fixed-site monitors, monitoring during yearly average concentration periods, or multiple measurement periods were used to estimate yearly averages [see Supplemental Material, Table 1 (doi:10.1289/ehp.1002976) for the city-specific data used for model evaluation]. These pollution measurements were collected at much higher spatial densities than were NAPS and from monitors that were located to specifically capture spatial pollutant gradients. Consequently, these data were reasonable for use as a gold standard to determine how well the two national NO_2_ and benzene models (the LUR models and the LUR models with gradients) predicted within-city variation. In addition, we compared the city-specific data with estimates based on inverse distance weighting (IDW) of annual average NO_2_ and benzene concentrations measured at NAPS monitors (with and without deterministic gradients). Because of NAPS monitor density in Canada, kriging could not be applied.

*Population exposure assessment.* The national pollutant models were applied to each of the 478,831 Statistics Canada street block-face centroid locations in 2006 to estimate population exposures. First, we applied the LUR models to each block point to derive a unique predicted pollutant concentration for each point, representing the average exposure level for 89 and a SD of ± 158 individuals. We used a GIS to identify the distance of each block centroid to the nearest highway, major road, local urban road, and gas stations and adjusted the corresponding LUR model estimate when the street block point was located within an associated gradient. We then estimated population-weighted exposures to PM_2.5_, NO_2_, benzene, ethylbenzene, and 1,3-butadiene in the Canadian population as a whole, and we estimated uncertainty using the 95% confidence limits for LUR model predictions. Because there was insufficient information in the literature to examine uncertainty for specific gradients, we selected ± 50% for all gradients (values shown in Table 1).

## Results

*National LUR model results.* Table 2 summarizes the national LUR model results. The PM_2.5_ model predicted 46% of PM_2.5_ variation and was dominated by satellite predictions, which alone explained 41% of PM_2.5_ variation. The NO_2_ model predicted 73% of NO_2_ variation and length of all roads within 10 km was the dominant predictor, explaining 55% of NO_2_ variation. This variable was only moderately correlated (*r* = 0.56) to NO_2_ predictions from satellite data, which further explained 4% of NO_2_ variation in the final model. The models for benzene, ethylbenzene, and 1,3-butadiene had similar predictive results, explaining 62, 67, and 68% of pollutant variability, respectively. Data from one monitor were removed as an outlier from the benzene and ethylbenzene models (St. John Baptiste, located in Montreal east city) and from the 1,3-butadiene model (Sarnia, located in southern Ontario near the Detroit–Windsor border), which were associated with the highest pollutant concentration for each substance.

**Table 2:** National LUR model results for PM_2.5_, NO_2_, benzene, ethylbenzene, and 1,3-butadiene.

Variable	Distance*a*	Value	SE	*p*-Value
PM_2.5_ model (*R*^2^ = 0.46, RMSE = 1.529)								
Intercept		—		2.802		0.497		< 0.0001
Satellite PM_2.5_ (µg/m^3^)		—		2.392		0.263		< 0.0001
NPRI emissions (tonnes)		5 km		1.63e–3		5.95e–4		0.007
Industrial land use (m^2^)		1 km		1.03e–6		4.18e–7		0.014
NO_2_ model (*R*^2^ = 0.73, RMSE = 5.470)								
Intercept		—		13.179		1.374		< 0.0001
Satellite NO_2_ (ppb)		—		1.4903		0.355		< 0.0001
Industrial land use (m^2^)		2 km		3.21e–6		5.73e–7		< 0.0001
Road length (m)		10 km		7.42e–6		9.04e–7		< 0.0001
Summer rainfall (mm)		—		–0.010		0.002		< 0.0001
Benzene model*b* (*R*^2^ = 0.62, RMSE = 0.298)								
Intercept		—		0.346		0.069		< 0.001
Major road length (m)		10 km		1.18e–6		2.56e–7		< 0.001
NPRI emissions (present)		10 km		0.526		0.089		< 0.001
Ethylbenzene model*c* (*R*^2^ = 0.67, RMSE = 0.193)								
Intercept		—		0.152		0.039		< 0.001
Population (count)		10 km		6.74e–7		7.25e–8		< 0.001
NPRI emissions (present)		2 km		0.272		0.071		< 0.001
1,3-Butadiene model*d* (*R*^2^ = 0.68, RMSE = 0.034)								
Intercept		—		0.011		0.009		0.208
Road length (m)		750 m		3.89e–6		7.93e–7		< 0.001
Highway (present)		500 m		0.041		0.012		0.002
Commercial land use (m^2^)		10 km		1.60e–9		5.97e–10		0.010
Satellite PM_2.5_ and NO_2_ are satellite-derived estimates of PM_2.5_ and NO_2_. Land use is the area of specific land-use types (industrial, commercial) within the associated buffer distance. Road length refers to the length of different road classifications (all, major, highways) within the associated buffer distance. Summer rainfall refers to the amount of rainfall recorded from May to September from the nearest meteorological station. NPRI emissions refer to the amount of annual emissions of the model substance released from industries that reported to the NPRI. NPRI emissions (present) refers to the presence of NPRI facilities that have released a model substance into the air. Population (count) refers to the number of individuals who resided within the associated buffer distance. **a**Radius of cicular buffers used to derive variables. **b**One outlier removed with benzene concentration of 3.55 µg/m^3^. **c**One outlier removed with ethylbenzene concentration of 2.57 µg/m^3^. **b**One outlier removed with 1,3-butadiene concentration of 0.82 µg/m^3^.								

Spatial autocorrelation of national LUR models. Spatial autocorrelation of the LUR model residuals was examined using Moran’s *I* in ArcGIS. Spatial autocorrelation was present in the PM_2.5_ LUR model residuals (Moran’s *I* = 0.33, *p* < 0.001), indicating that a moderate amount of spatial autocorrelation remained that was not explained by the PM_2.5_ model predictors. Clustering of positive residuals (model underpredicting by an average of 2.57 µg/m^3^) occurred in the rural interior of British Columbia. An indicator variable for British Columbia substantially reduced the spatial autocorrelation (Moran’s *I* = 0.03, *p* = 0.04). Sensitivity analysis using a summer-only PM_2.5_ model indicated no spatial autocorrelation (Moran’s *I* = 0.04, *p* = 0.01), supporting our hypothesis of woodburning as the primary source of model underprediction in this region. No significant spatial autocorrelation existed in LUR model residuals for NO_2_ (Moran’s *I* = 0.03, *p* = 0.44), benzene (Moran’s *I* = –0.20, *p* = 0.13), ethylbenzene (Moran’s *I* = –0.00, *p* = 0.87), and 1,3-butadiene (Moran’s *I* = 0.09, *p* = 0.32).

**Figure 2:**
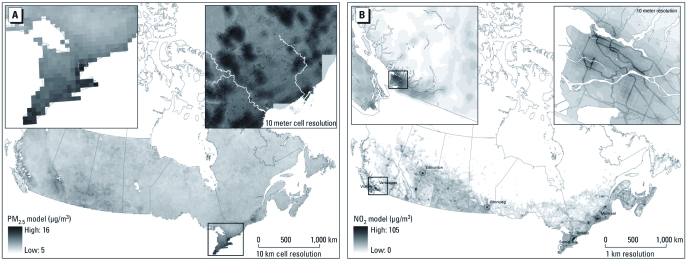
National annual average models for PM_2.5_, highlighting southern Ontario and the city of Toronto (*A*), and for NO_2_, highlighting southwestern British Columbia and the city of Vancouver (*B*), that incorporate satellite-derived pollutant estimates, geographic land use variables, and deterministic gradients. The seven cities shown in (*B*) represent locations of independent monitoring data used to evaluate the national NO_2_ and benzene models.

**Figure 3:**
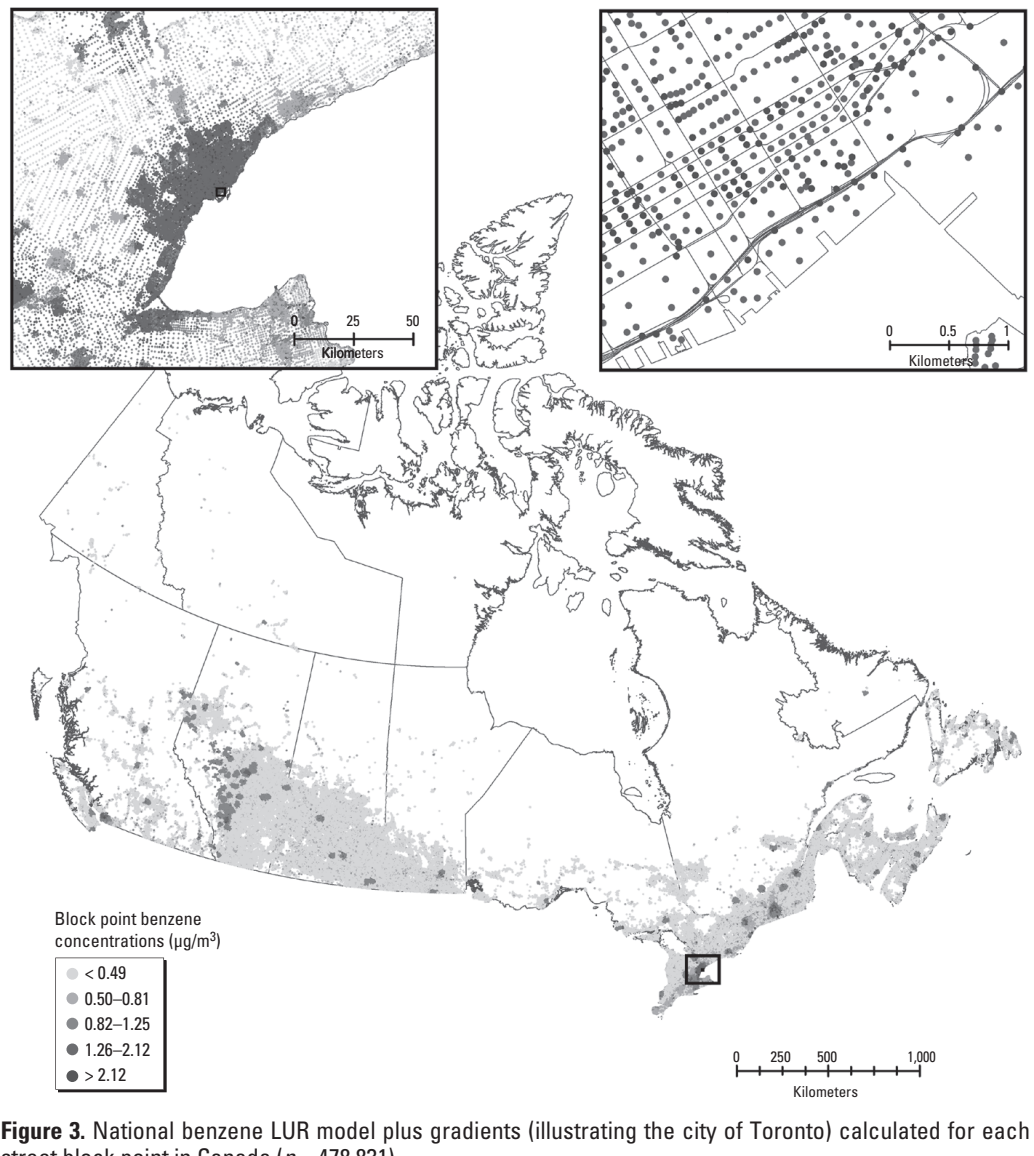
National benzene LUR model plus gradients (illustrating the city of Toronto) calculated for each street block point in Canada (*n* = 478,831).

*Incorporating gradients with national LUR models.* Deterministic gradients were added to LUR models, because we could not estimate the effects of local-scale pollution sources from NAPS data alone. Figure 2A illustrates the final PM_2.5_ model (LUR plus gradients) for Canada as a whole and for southern Ontario and the city of Toronto. Figure 2B illustrates the final national NO_2_ model (LUR plus gradients) for Canada as a whole and for southwestern British Columbia and the city of Vancouver. These maps illustrate the spatial resolution of the final national pollutant models; however, for population exposure assessment, the LUR model results and deterministic gradients were applied to street block point locations, as shown in Figure 3, which illustrates the final national benzene model (LUR plus gradients) calculated at the block point level.

*Evaluation of national pollutant models.* Evaluation of LUR models using bootstrap and leave-one-out analyses. The distribution of all model coefficients for each pollutant resulting from bootstrap analysis showed normal distributions. The NO_2_ model was the least sensitive to monitor selection, with a bootstrap *R*^2^ 95% CI of 65–81. Models for PM_2.5_, benzene, ethylbenzene, and 1,3-butadiene demonstrated larger uncertainty to monitor selection, with *R*^2^ 95% CIs of 33–59, 44–80, 49–85, and 53–82, respectively. Variable coefficients for industrial NPRI proximity variables were extremely sensitive to monitor selection. The leave-one-out analyses indicated no significant bias in any LUR model, as demonstrated by the mean ± SD error: 1.07e–3 ± 5.61 for NO_2_; –6.35e–3 ± 1.59 for PM_2.5_; –0.04 ± 0.32 for benzene; –0.01 ± 0.04 for 1,3-butadiene; and –0.04 ± 0.22 for ethylbenzene.

Evaluation of NO_2_ and benzene models using city-specific data. On average, the national NO_2_ LUR plus gradient model predicted 43% of the within-city NO_2_ variation (based on the city-specific data evaluation) compared with 22% predicted based on IDW of NAPS monitors plus gradients (Table 3). National LUR, LUR plus gradients, IDW, and IDW plus gradients models overpredicted the city-specific NO_2_ measurements, with average city-specific intercepts of 4.56, 7.45, 8.51, and 11.56 µg/m^3^, respectively. City-specific scatter plots of measured and modeled NO_2_ concentrations are illustrated in Supplemental Material, Figure 1 (doi:10.1289/ehp.1002976).

**Table 3:** Evaluation of national NO_2_ and benzene models, as well as IDW estimates from fixed-site monitors, against independent city-specific measurement data.

*R*^2^ (RMSE)						
Substance	*n^a^*	LUR*^b^*	LUR + G*^c^*	IDW*^d^*	IDW + G*^e^*
NO_2_										
Edmonton		50		0.60 (3.67)		0.41 (4.59)		0.10 (5.52)		0.01 (5.92)
Montreal		135		0.41 (4.28)		0.48 (4.04)		0.31 (4.63)		0.41 (4.29)
Sarnia		34		0.42 (4.21)		0.49 (4.04)		0.12 (5.15)		0.19 (5.12)
Toronto		196		0.18 (7.69)		0.36 (6.78)		0.13 (7.93)		0.32 (6.99)
Victoria		40		0.19 (3.95)		0.37 (3.70)		0.23 (3.86)		0.26 (3.98)
Vancouver		114		0.31 (6.41)		0.42 (5.93)		0.31 (6.43)		0.36 (6.24)
Winnipeg		49		0.54 (3.65)		0.51 (3.86)		0.08 (5.17)		0.02 (5.43)
Average		618		0.39 (4.84)		0.43 (4.71)		0.18 (5.53)		0.22 (5.42)
Benzene										
Montreal*f*		131		0.33 (0.24)		0.26 (0.25)		0.11 (0.28)		0.05 (0.29)
Sarnia		37		0.02 (0.57)		0.04 (0.56)		0.00 (0.57)		0.03 (0.56)
Toronto*g*		44		0.03 (0.19)		0.22 (0.17)		0.00 (0.19)		0.34 (0.16)
Winnipeg		94		0.08 (0.25)		0.10 (0.25)		0.00 (0.26)		0.01 (0.26)
Average		306		0.12 (0.31)		0.16 (0.31)		0.03 (0.33)		0.11 (0.32)
**a**Number of within-city measurement locations. **b**National LUR model. **c**National LUR model plus gradients (G). **d**IDW interpolation of NAPS fixed-site monitoring data. **e**IDW interpolation of NAPS fixed-site monitoring data plus gradients. **f**Four outliers removed with highest city concentrations (> 2 µg/m^3^). **g**One outlier removed with highest city concentration (4.10 µg/m^3^).										

For benzene, all modeling methods performed poorly in explaining within-city benzene variation. The LUR plus gradients model explained, on average, only 16% of within-city variability in benzene concentrations compared with 11% based on IDW plus gradients (Table 3). In the evaluation using the Montreal city-specific benzene concentrations, four outliers were removed (all concentrations > 2 µg/m^3^), and one outlier (4.10 µg/m^3^) was removed in the Toronto evaluation. Benzene models also overpredicted city-specific concentrations, based on city-specific intercepts of modeled versus measured concentrations [see Supplemental Material Figure 2 (doi:10.1289/ehp.1002976)]. Sarnia, a high-density industrial community with 46 NPRI emitters, had poor NO_2_ and benzene model evaluations.

*Canadian population exposure assessment.* The final LUR models and gradients were applied to all 478,831 street block-face centroid locations to conduct population exposure assessments. Estimated mean (95% CI) population exposures (micrograms per cubic meter) to ambient PM_2.5_, NO_2_, benzene, ethylbenzene, and 1,3-butadiene in Canada based on the LUR models were 8.10 (5.84–10.43), 22.40 (13.14–33.51), 0.94 (0.57–1.31), 0.38 (0.25–0.52), and 0.086 (0.035–0.138), respectively. Estimates for the same pollutants based on the national LUR plus gradients models were 8.39 (6.00–11.13), 23.37 (14.01–35.73), 1.04 (0.59–1.49), 0.63 (0.35–1.10), and 0.089 (0.036–0.146), respectively. Wide ranges of exposure levels were estimated in Canada for all substances; see Supplemental Material, Figure 3 (doi:10.1289/ehp.1002976) for population exposure distributions.

## Discussion

We created national pollutant models from fixed-site monitoring data that incorporate satellite, geographic, and deterministic components and demonstrated that these models can improve exposure assessment over large geographic areas compared with approaches based solely on interpolation of fixed-site monitoring data. We also demonstrated how these models can be used for population exposure assessment.

The national LUR models explained 73% of pollution variation in NAPS measurements for NO_2_, and lesser degrees for PM_2.5_ (46%), benzene (62%), ethylbenzene (67%), and 1,3-butadiene (68%). The NO_2_ and PM_2.5_ models were least sensitive to monitor selection, whereas models for VOCs were more sensitive—likely because of the smaller number of monitors on which LUR estimates were based (*n =* 53). The predictive performance of the PM_2.5_ model [*R*^2^ = 0.46, root mean square error (RMSE) = 1.53 µg/m^3^] was consistent with other large-scale modeling studies based on different monitoring methodologies and data inputs (Beelen et al. 2009; Hart et al. 2009; Liao et al. 2006; Ross et al. 2007).

The national LUR models generally captured regional patterns in pollutant concentrations, corresponding to NAPS monitor siting criteria, but were less effective at identifying small-scale geographic predictor variables. For example, only 35 NAPS monitors were located within 500 m of a major road and only 7 monitors were within 500 m of a major industrial emission source. Such small sample sizes greatly reduce the power of the models to capture these specific pollutant sources. Some city-specific LUR methods have used location-allocation methods to more fully represent the true spatial variation in pollution levels and to capture the range of predictor variables (Jerrett et al. 2005). Models based on fixed-site monitor data may therefore need additional approaches to represent local-scale pollutant variability not captured by fixed-site monitors. This was indeed the case with the Canadian NAPS network, but larger regulatory networks, such as those in the United States, may better represent the range of predictor variables needed to build local-scale LUR models.

To address the lack of local-scale geographic variability in the NAPS data, we incorporated deterministic gradients based on proximity to specific sources (i.e., vehicles and gas stations). The final NO_2_ LUR plus gradient model improved prediction of within-city pollutant variation considerably compared with the LUR model alone and interpolation methods. On average, the final model predicted 43% of within-city NO_2_ variation compared with 18% using IDW. Both the national benzene model and IDW predicted within-city benzene poorly, which may be due to the small number of NAPS monitors on which the model was based, the relatively small variation in within-city benzene levels, or the inability of gradients to capture local benzene concentrations. Similar to the NO_2_ model, the evaluation of the benzene model with Sarnia data was poor, reflecting the difficulty in capturing unique high-density industrial conditions in a national model.

Gradients were based on literature reviews. The lack of methodological consistency among published data of pollutant level increases near specific sources and the distance required for pollutant levels to return to background were clear limitations. To improve reliability of gradients, we used linear functions to represent the decreases in pollutant levels found in the initial portions of the exponential decay curves found in the literature. The methodology used here could be augmented as new gradients become available or with other modeled data.

Population exposure assessment was conducted using the national models and census street block-face points. The population-weighted average exposures to PM_2.5_, NO_2_, benzene, ethylbenzene, and 1,3-butadiene were 8.39, 23.37, 1.04, 0.63, and 0.089 (µg/m^3^), respectively. The uncertainty of population exposure estimates were driven primarily by LUR model uncertainty. Although the results of the national LUR models are similar to city-specific LUR models in their predictive capacity and error, we are unaware of any LUR models that have been applied to estimate exposure uncertainty. Although these exposures are low compared with other developed countries, exposures in particular locations in Canada are relatively high. For example, the 90th percentiles of exposures (micrograms per cubic meter) are 9.78 for PM_2.5_, 34.81 for NO_2_, 1.61 for benzene, 1.01 for ethylbenzene, and 0.14 for 1,3-butadiene. The ability of the national models to capture local-scale pollutant variability allows for more realistic exposure assessments and assessments that can potentially identify high-risk populations. Future work will refine approaches for using the national models to calculate population exposure assessments, incorporate socioeconomic information from census to examine environment injustice issues, and integrate national models into a risk-assessment framework that incorporates exposures from other sources and microenvironments.

This study faced a number of challenges and limitations to creating national pollutant models from fixed-site monitors and applying these models to estimate Canadian population exposures. First, the NAPS monitors in Canada are centered in large metropolitan areas, and modeled relationships will therefore be weighted toward these areas. This is appropriate for population exposure assessment, because these locations represent the majority of Canadians, but in rural areas the models could be adjusted or a background concentration could be used. This is particularly relevant to the benzene, ethylbenzene, and 1,3-butadiene models, which were based on data from monitors located almost exclusively in large urban areas or sited near large industrial sources. Second, we had limited data on pollutant sources and source strengths such as traffic volumes. In addition, we did not model emissions from woodburning stoves and forest fires, which may have caused us to underpredict PM_2.5_ concentrations in the interior of British Columbia. Third, parsimonious LUR models were created, because the specificity of model variables may be important for informing surveillance and regulation. This, however, leads to models that do not capture the complex interactions between geographic characteristics and pollutant sources, and even the simplest LUR predictors (e.g., major roads or NPRI facilities within 10 km) capture complex mixes of geographic characteristics and pollutant sources. Fourth, we compared model estimates with city-specific measurements for NO_2_ and benzene collected in different years and using a variety of methodologies. Nevertheless, these measurements represent the best data on within-city pollutant variability available. Fifth, applying LUR model results to approximately half a million block points is currently extremely computationally and time intensive. Finally, the geographic accuracy of street block centroids may introduce errors into the gradient portions of the models and therefore the exposure assessment, particularly between rural and urban areas. These errors, however, are likely spatially random within rural and urban areas across Canada.

## Conclusions

National exposure models were required by Carex Canada to produce population exposure assessments that captured both between-city and within-city pollution variability. We created national PM_2.5_, NO_2_, benzene, ethylbenzene, and 1,3-butadiene models from fixed-site monitoring data and found that a combination of data sources and methods to capture background, regional, and local-scale pollution variation improved exposure assessment over traditional IDW interpolation approaches. The national pollutant models were applied to street block-face points, representing the locations of the Canadian population, to determine population exposure estimates. Estimates of average population exposure levels in Canada are PM_2.5_ 8.39, NO_2_ 23.37, benzene 1.04, ethylbenzene 0.63, and 1,3-butadiene 0.09 (µg/m^3^). The modeling approach developed here uses readily available data and could be reproduced over time, for example, every 5 years with the Canadian census. This would provide updated population exposure assessments and a long-term surveillance capacity for monitoring trends in population exposures, for identifying potential susceptible populations and geographic locations with elevated exposures, and for evaluating the impacts of policies and regulatory changes on exposure levels.

## Supplemental Material

(80 KB) PDF
