# Supplementary material for: Creating National Air Pollution Models for Population Exposure Assessment in Canada
Source: Environ Health Perspect. 2011 Mar 31;119(8):1123–9. doi: 10.1289/ehp.1002976 (PMC3237350; doi:10.1289/ehp.1002976)
Supplement: (80 KB) PDF [file ehp.1002976.s001.pdf]

SUPPLEMENTAL MATERIALS

Creating National Air Pollution Models for Population Exposure Assessment in Canada

Perry Hystad[1], Eleanor Setton[2], Alejandro Cervantes[3], Karla Poplawski[4], Steeve Deschenes[2], Michael Brauer[4], Aaron van Donkelaar[5], Lok Lamsal[5], Randall Martin[5,6], Michael Jerrett[7], Paul Demers[4,8]

1. School of Population and Public Health, University of British Columbia, Vancouver, BC, Canada
2. Department of Geography, University of Victoria, Victoria, BC, Canada
3. Department of Geography, University of British Columbia, Vancouver, BC, Canada
4. School of Environmental Health, University of British Columbia, Vancouver, BC, Canada
5. Department of Physics and Atmospheric Science, Dalhousie University, Halifax, Nova Scotia, Canada
6. Harvard-Smithsonian Center for Astrophysics, Cambridge, Massachusetts, USA
7. School of Public Health, Division of Environmental Health Science, University of California, Berkeley, California, USA
8. Occupational Cancer Research Centre, Cancer Care Ontario

Table of Contents:

Supplemental Material, Table 1. Summary statistics of monitoring data collected from previous city-specific LUR monitoring in Canada and used to evaluate national NO_2 and benzene models — Page 2

Supplemental Material, Figure 1. Evaluation of national NO_2 model, incorporating satellite data, geographic landuse variables and deterministic gradients, with independent within-city measurements. — Page 3

Supplemental Material, Figure 2. Evaluation of national Benzene model, incorporating geographic landuse variables and deterministic gradients, with independent within-city measurements. — Page 4

Supplemental Material, Figure 3. Annual 2006 Canadian population exposure estimates from national LUR plus gradient models (Frequencies represent street block points, each containing approximately 89 individuals). — Page 5

Supplemental Material, Table 1. Summary statistics of monitoring data collected from previous city-specific LUR monitoring in Canada and used to evaluate national NO_2 and benzene models.

Substance[a]	Year	N	Mean +/-SD	Min	Max
NO_2(µg/m^3)					
Edmonton[b]	2008	50	28.90 (5.88)	17.09	42.96
Montreal[c]	05/06	135	22.00 (5.77)	7.44	35.81
Sarnia[d]	2005	35	19.66 (6.28)	2.39	31.41
Toronto[d,e]	04/06	196	24.20 (8.46)	8.98	52.18
Victoria[b]	2006	40	9.49 (4.87)	0.75	19.18
Vancouver[b]	2003	114	30.08 (7.76)	14.49	52.64
Winnipeg[b]	2008	49	16.06 (5.43)	4.25	32.98
Benzene(µg/m^3)					
Montreal[c]	05/06	135	1.05 (0.44)	0.39	3.35
Sarnia[d]	2005	37	0.93 (0.56)	0.28	3.36
Toronto[d]	2006	45	0.75 (0.54	0.40	4.10
Winnipeg[b]	2008	94	0.44 (0.26)	0.08	1.12

[a]Each city conducted monitoring for 2 week periods.
[b]Fixed site monitoring was used to adjust to yearly average.
[c]Average of 3 seasons of monitoring (Dec., May., Aug.) used to capture yearly average.
[d]No yearly adjustment conducted (Sarnia data collected in October and Toronto in September).
[e]Combined 2004 and 2006 data for evaluation.

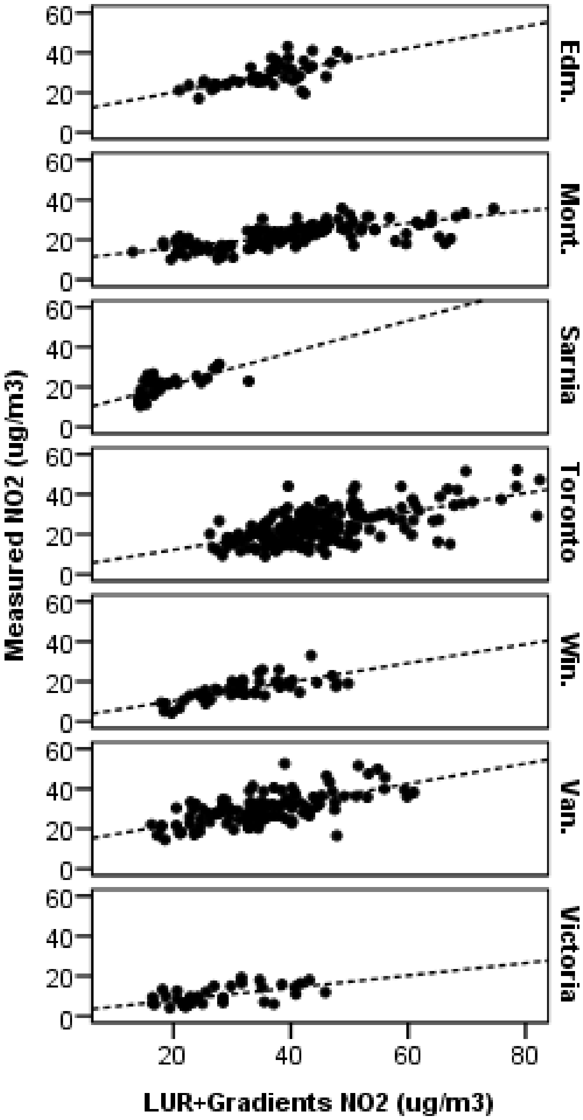

Supplemental Material, Figure 1. Evaluation of national NO_2 model, incorporating satellite data, geographic landuse variables and deterministic gradients, with independent within-city measurements.

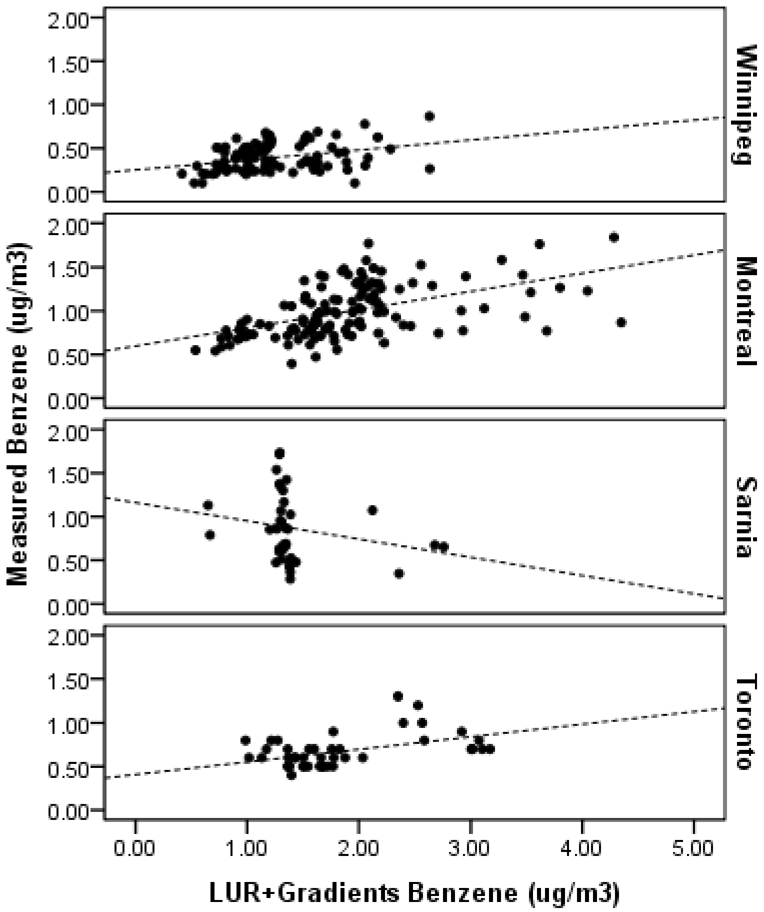

Supplemental Material, Figure 2. Evaluation of national Benzene model, incorporating geographic landuse variables and deterministic gradients, with independent within-city measurements.

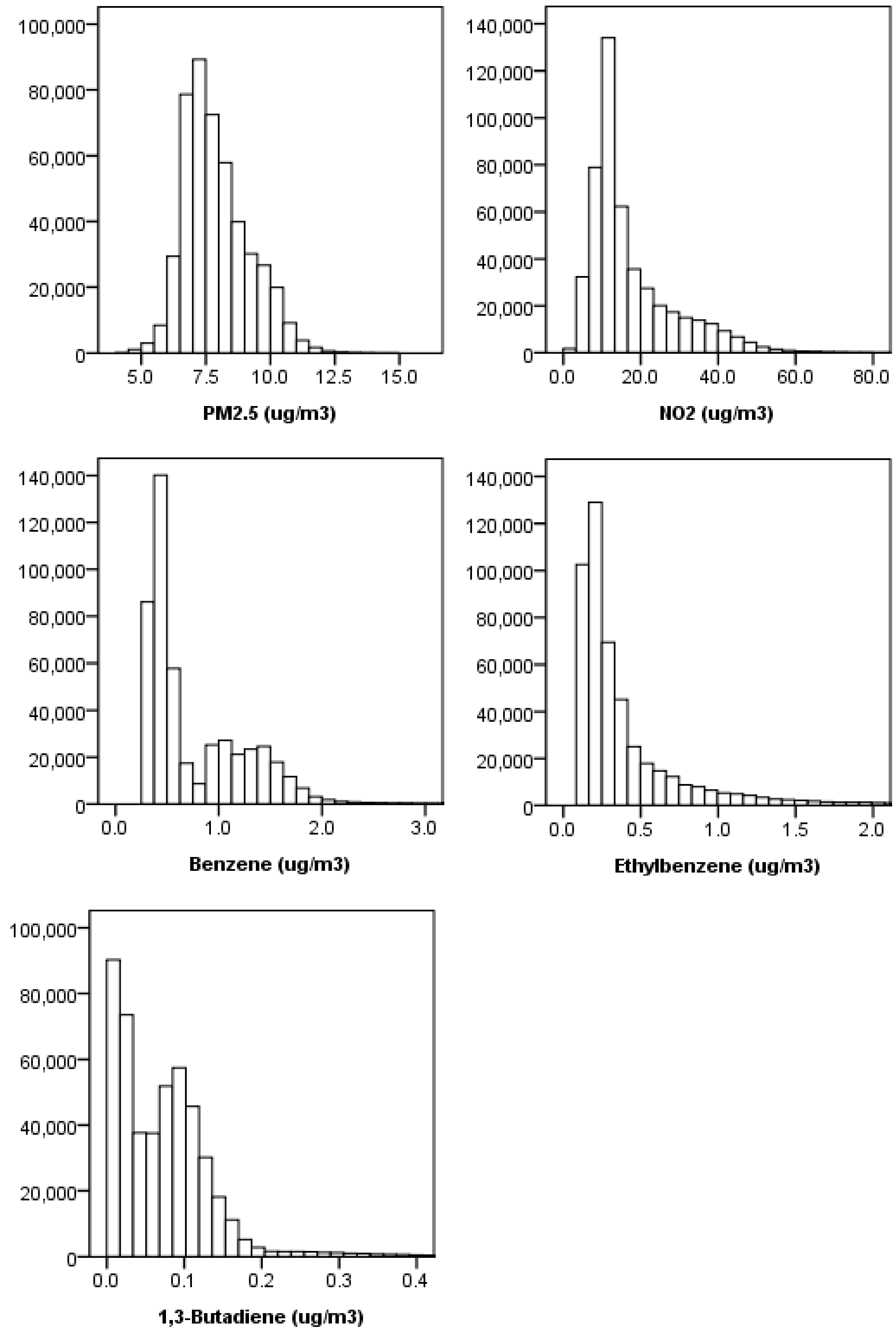

Supplemental Material, Figure 3. Annual 2006 Canadian population exposure estimates from national LUR plus gradient models (Frequencies represent street block points, each containing approximately 89 individuals).
